# Expression of Genes Involved in Heavy Metal Trafficking in Plants Exposed to Salinity Stress and Elevated Cd Concentrations

**DOI:** 10.3390/plants9040475

**Published:** 2020-04-09

**Authors:** Michał Nosek, Adriana Kaczmarczyk, Roman J. Jędrzejczyk, Paulina Supel, Paweł Kaszycki, Zbigniew Miszalski

**Affiliations:** 1 Institute of Biology, Pedagogical University, Podchorążych 2, 30-084 Kraków, Poland; 2 The Franciszek Górski Institute of Plant Physiology Polish Academy of Sciences, Niezapominajek 21, 30-239 Kraków, Poland; a.kapron@ifr-pan.edu.pl (A.K.); z.miszalski@ifr-pan.edu.pl (Z.M.); 3 Małopolska Centre of Biotechnology, Jagiellonian University, Gronostajowa 7a, 30-387 Kraków, Poland; roman.jedrzejczyk@uj.edu.pl; 4 Department of Plant Biology and Biotechnology, Faculty of Biotechnology and Horticulture, University of Agriculture in Krakow, al. 29 Listopada 54, 31-425 Kraków, Poland; paulina.supel@urk.edu.pl (P.S.); pawel.kaszycki@urk.edu.pl (P.K.)

**Keywords:** heavy metal stress, heavy metals transporters, *Mesembryanthemum crystallinum*, phytoremediation, semi-halophyte

## Abstract

Many areas intended for crop production suffer from the concomitant occurrence of heavy metal pollution and elevated salinity; therefore, halophytes seem to represent a promising perspective for the bioremediation of contaminated soils. In this study, the influence of Cd treatment (0.01–10.0 mM) and salinity stress (0.4 M NaCl) on the expression of genes involved in heavy metal uptake (*irt2*–iron-regulated protein 2, *zip4*–zinc-induced protein 4), vacuolar sequestration (*abcc2*–ATP-binding cassette 2, *cax4*–cation exchanger 2 *pcs1*–phytochelatin synthase 1) and translocation into aerial organs (*hma4*–heavy metal ATPase 4) were analyzed in a soil-grown semi-halophyte *Mesembryanthemum crystallinum*. The upregulation of *irt2* expression induced by salinity was additionally enhanced by Cd treatment. Such changes were not observed for *zip4*. Stressor-induced alterations in *abcc2*, *cax4*, *hma4* and *pcs1* expression were most pronounced in the root tissue, and the expression of *cax4*, *hma4* and *pcs1* was upregulated in response to salinity and Cd. However, the cumulative effect of both stressors, similar to the one described for *irt2*, was observed only in the case of *pcs1*. The importance of salt stress in the *irt2* expression regulation mechanism is proposed. To the best of our knowledge, this study is the first to report the combined effect of salinity and heavy metal stress on genes involved in heavy metal trafficking.

## 1. Introduction

Among abiotic stresses, salinity is one of the most important factors affecting crop growth. According to the FAO Land and Plant Nutrition Management Service, elevated salinity concerns more than 6% of the land area (approximately 400 million hectares) and increases by approximately 1–2% a year. This phenomenon is predominantly associated with human activities, such as excessive irrigation, deforestation, fertilization and irrigation with salt-contaminated water, resulting in poor drainage, elevated water table, waterlogging and salt accumulation in the root zone [1,2,3,4]. Even good quality water used for irrigation may contain 200–500 mg of soluble salts per litre. According to [2], a hectare of arable land may receive 3–5 tons of salts per year in this way. Salinity stress can be recognized by the occurrence of three symptoms: first, increased osmotic pressure, which results in water deficit conditions; second, accumulation of toxic ions in plant organs [4]; and third, nutritional imbalance, which influences the growth and development of challenged plants [5]. These symptoms are primarily responsible for adverse alterations in morphological, physiological and biochemical processes that disrupt the agricultural production and ecological balance of the area [3].

Many areas, including arable lands, suffer from concomitant contamination with heavy metals (HMs) and salts. Recently, the accumulation of HMs in the environment has increased as a consequence of intensified human activities, such as mining, smelting and agricultural practices, including the long-term application of fertilizers, fungicides or pesticides [6]. Since HMs are non-degradable, they may persist in a contaminated substrate for decades [7]. The threat posed by heavy metals to humans has an origin in plants, which are the first link in the food chain. Plants can take up HMs from various sources, such as air, water or soil; the latter process predominates and is dependent on many factors, such as temperature, soil pH, aeration and, clearly, on the particular species involved in the accumulation process.

Cadmium is one of the HMs that is particularly widely spread in the environment and has gained prominence as an important issue not only in urbanized lands, but also in less explored, rural, agricultural or wildlife areas. Cd toxicity results from altered uptake and transport of nutrient elements (Ca, Mg, P), disruption of water balance, inhibition of enzymes and reactive oxygen species (ROS) overproduction. As a consequence, sensitive plants growing in the Cd-polluted environment display such symptoms as chlorosis, growth inhibition and root tip browning [8]. Since plant responses to salinity and HM stresses rely on common physiological mechanisms, halophytes have been proposed as potentially useful candidates for the phytoremediation of Cd-contaminated saline soils [7,9,10,11]. Halophytes are the plants capable of growth and able to complete their life cycle in environments polluted with high salt concentrations [2]. Although these plants represent only 2% of the total plant species on Earth, they are a highly diverse group of organisms [12]. The high tolerance of halophytes towards salinity is a result of specific properties at the molecular, cellular and whole-plant levels, and is at least partially related to enhanced resistance to oxidative stress [2,5]. In addition to effective scavenging of salt stress-derived ROS, halophytes display a whole range of various mechanisms, including the development of special tubers at leaves or stems that may contain large amounts of salt, the production of enzymes that limit ion transfer through membranes, the generation of stress proteins and osmolytes and active metal detoxification in vacuoles, which enable homeostasis maintenance under stressful conditions [11].

Halophytic plants employ many strategies enabling the undisturbed execution of the developmental program under concurrent salinity and HM stresses. These strategies involve restricted metal uptake, exclusion of toxic ions from roots, efficient neutralization of metal ions in the protoplast and sequestration or translocation to remote organs [13]. The above mechanisms are regulated by a large number of genes and provide higher tolerance to both salt and Cd [5]. The members of the zinc iron permease (ZIP) family, including iron-regulated transporters (IRT), are responsible for zinc (Zn), manganese (Mn), iron (Fe) and Cd uptake [14]. Physiological studies confirmed the overexpression of ZIP family genes in plants exposed to Cd and nickel (Ni) [15]. Among the transporters involved in Cd trafficking, phytochelatins (PCs) are the best-known protoplast chelating agents required for the maintenance of Cd tolerance. Sequestration of Cd in vacuoles is a well-described process directing plant tolerance towards this pollutant, which involves at least a few known transporters. Cation exchangers (CAX) participate in Cd^2+^/Na^+^ antiport activity in tonoplasts exploiting a V-H^+^-ATPase-derived proton motive force [15]. In addition to CAXs, the C subfamily of the ATPase binding cassette (ABCC) has been recognized as an additional vacuolar metal-PC transporter. Although it has been reported that vacuolar sequestration of Cd by ABCC is essential for its complete detoxification, transporters from this subfamily are also involved in the transport of such ions as Zn, Cu, Mn and Fe [16]. Apart from these strategies, the translocation of Cd to remote organs, combined with sequestration in tissues not involved in vital metabolic processes, seems to play a crucial role in Cd tolerance. Effective translocation of Cd to the shoots requires active transport during loading to the xylem. The P-type heavy metal ATPases (HMA) are transporters involved both in Cd loading to xylem from vascular tissues (HMA4) [17]. Regulation of the expression of genes associated with the presented transporters may be necessary for the appropriate distribution of toxic metal ions at the cellular and whole-plant levels, which substantially contributes to the resultant Cd tolerance in plants [15].

Many halophytes were reported to be involved in HM remediation processes [10,18,19,20]. One of them is *Mesembryanthemum crystallinum* (the common ice plant), the model C_3_-CAM intermediate semi-halophyte, which is widely studied in physiological and biochemical research. This plant has been found to reveal a high tolerance to excess light, drought and salinity stresses. The dry weight of the plant may contain even 40% NaCl without showing visible symptoms of toxicity [9]. In addition to salinity tolerance, high resistance towards HMs has recently been confirmed. This halophyte is able to accomplish its life cycle at high concentrations of Cu and Zn present in the soil substrate. It was also confirmed that high doses of Ni did not induce any growth or developmental disturbances [9,21,22]. In our studies, we described high tolerance of soil-grown ice plants towards elevated Cd levels, regardless of the photosynthetic metabolism [23]. The obtained data strongly suggest the potential use of *M. crystallinum* in the phytoremediation of HM-polluted areas. The aim of the present study was to determine expression alterations of transporters involved Cd uptake (*zip4*, *irt2*), protoplast detoxification (*pcs1*), vacuolar sequestration (*abbc2*, *cax4*) and xylem loading/shoot translocation (*hma4*) upon salinity and heavy metal stress in a model semi-halophyte known for enhanced HM resistance. To the best of our knowledge, this study is the first to describe the impact of combined stresses, i.e., salinity and HM, on the expression of genes involved in heavy metals trafficking.

## 2. Results

### 2.1. Cadmium Treatment up to 10 mM Does Not Affect the Growth and Development of Soil-Grown Common Ice Plants

In our previous paper, we showed that Cd concentrations up to 1 mM had no detrimental effects on soil-grown ice plants [23]. In this study, with the treatment extended to a Cd concentration of 10 mM, no visible morphological symptoms of heavy metal stress in either NaCl-untreated (−NaCl) or salt-stressed (+NaCl) plants were observed. In particular, none of the applied treatments significantly modified the dry weight of roots and shoots of NaCl-untreated and salt-stressed ice plants (Table 1). The water status of the roots and shoots of the −NaCl and +NaCl ice plants was not altered by any of the applied Cd concentrations (Table 2). Moreover, the unaffected plant growth was also confirmed by maintaining a constant shoot-to-root (DW) ratio, which demonstrated that neither root nor shoot growth was disrupted by Cd presence (Table 3). Taken together, the results obtained through biometric analysis have demonstrated high tolerance of both plant groups towards Cd treatments of up to 10 mM.

### 2.2. NaCl-Stressed Plants Accumulated More Cd than NaCl-Untreated Plants; Cd Was Deposited mostly in the Roots

**Figure 1:**
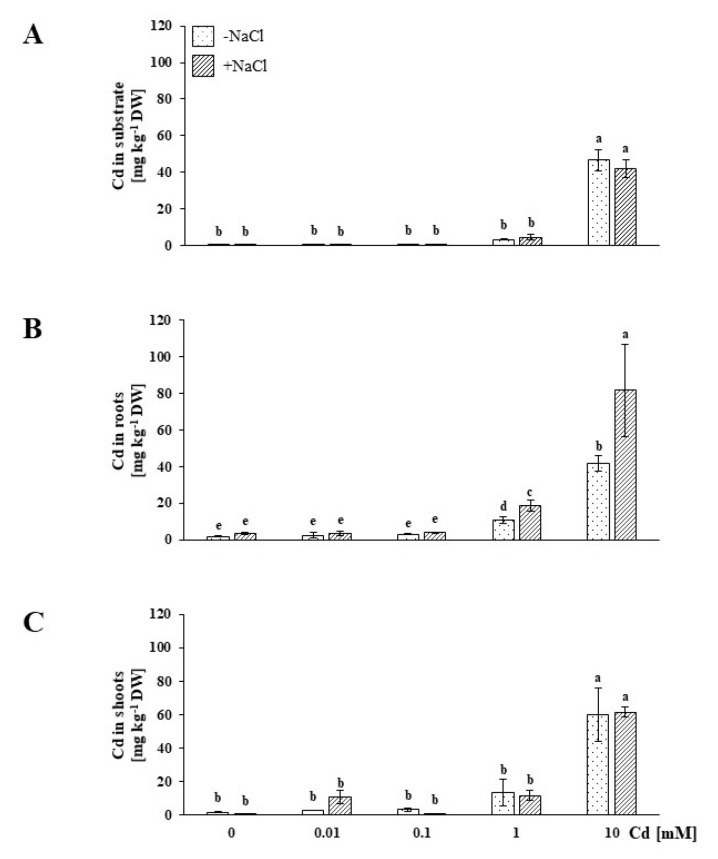
Contents measured in the substrate (**A**), roots (**B**) and shoots (**C**) of soil-grown NaCl-untreated (−NaCl) and salt-stressed (+NaCl) *Mesembryanthemum crystallinum* plants subjected to concentrations of 0.01, 0.1, 1, 10 mM Cd and the control (0.0 mM). Different letters above the bars indicate statistically significant differences at *p* ≤ 0.05 by Duncan’s post hoc test (*N* = 4, mean value ± SD).

To determine the concentration of Cd fraction available for the plants during the experiment, the substrate samples were analyzed with atomic absorption spectrometry (AAS). Detailed results regarding changes in bioavailable Cd concentration during subsequent days of treatment are presented in the supplementary data set (Figure S1). It is worth mentioning that, upon administration of the highest Cd treatment (10 mM), the salt-stressed (+NaCl) plants, beginning on the first day of the experiment, were exposed to a significantly higher concentration of bioavailable Cd in comparison to the NaCl-untreated plants. According to our preceding study concerning the *M. crystallinum*-Cd interaction [23], we focused on the results of the eighth day of treatment. In this study, we confirmed our previous observations by showing that with up to 0.1 mM treatment, the amount of bioavailable Cd was very low (Figure 1A). The low heavy metal bioavailability in the substrate was reflected by low Cd concentrations measured in corresponding root and shoot samples (Figure 1B,C). On the other hand, we found that under 1 and 10 mM Cd treatment, both −NaCl and +NaCl plants were exposed to similar concentrations of bioavailable Cd. As in our earlier work, the salt-stressed plants subjected to a 1 mM concentration accumulated more Cd compared to the NaCl-untreated plants, and the heavy metal was stored mainly in the roots (Figure 1B). Interestingly, this relationship was also maintained under 10 mM Cd treatment, where the salt-stressed plant roots accumulated almost two-fold more Cd in comparison to the roots of NaCl-untreated plants. The elevated Cd amounts were deposited in shoots of NaCl-untreated and salt-stressed plants only under 10 mM concentration, without distinctive differences being observed between both plant groups.

### 2.3. Genes Involved in Divalent Cation Uptake, Vacuolar Sequestration and Translocation Are Upregulated in the Roots of Plants Exposed to Salinity Stress

**Figure 2:**
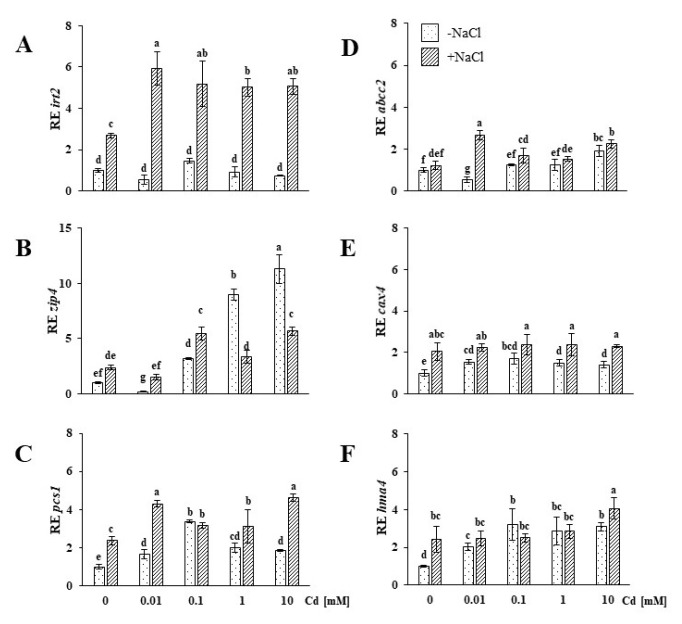
Expression of iron-regulated protein 2, *irt2* (**A**), zinc-induced protein 4, *zip4* (**B**), phytochelatin synthase 1, *pcs1* (**C**), ATP-binding cassette type c 2, *abcc2* (**D**), cation exchanger 4, *cax4* (**E**), and heavy metal ATPase 4, *hma4* (**F**) genes in the roots of soil-grown NaCl-untreated (−NaCl) and salt-stressed (+NaCl) *Mesembryanthemum crystallinum* plants subjected to concentrations of 0.01, 0.1, 1, 10 mM Cd and control (0.0 mM). Different letters above the bars indicate statistically significant differences at *p* ≤ 0.05 by Duncan’s post hoc test (*N* = 4, mean value ± SD).

To determine how salinity stress occurrence and/or Cd exposure affect the expression of the genes involved in uptake, vacuolar sequestration and aerial organ translocation, corresponding transcript levels were analysed with a qPCR technique in roots and shoots of the NaCl-untreated and salt-stressed plants exposed to elevated Cd concentrations. Expression of *irt2* and *zip4*, i.e., plasma membrane transporters involved in divalent cation uptake, were measured exclusively in the roots of both plant groups. The expression of *irt2* found in the Cd-untreated +NaCl plants was over two-fold higher in comparison to the control (Cd-, NaCl-untreated plants, Figure 2A). Cd treatment caused a greater than two-fold increase in *irt2* expression in the roots of salt-stressed plants, contrary to −NaCl roots, where none of the applied Cd treatments altered the gene expression. In the case of *zip4*, no significant difference was found between the control and salt-stressed plants (Figure 2B). The lowest Cd concentration (0.01 mM) caused *zip4* downregulation in the NaCl-untreated plants, while in the salt-stressed plants, the expression of this gene remained unaltered. Upon treatment with 0.1 mM Cd, *zip4* was upregulated in both groups; however, the concentration-dependent tendency to increase expression was maintained only in the −NaCl plants. In the roots of the salt-stressed (+NaCl) plants, treatment with 1 and 10 mM Cd resulted in similar *zip4* upregulation.

**Figure 3:**
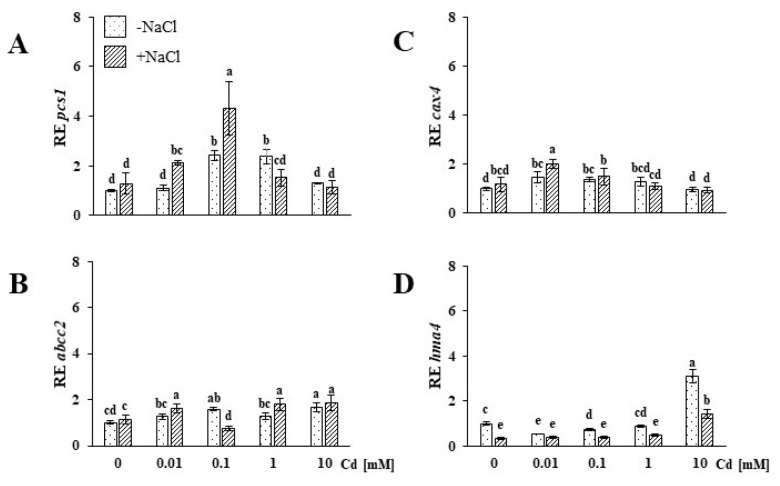
Expression of phytochelatin synthase 1, *pcs1* (**A**), ATP-binding cassette type c 2, *abcc2* (**B**), cation exchanger 4, *cax4* (**C**), and heavy metal ATPase 4, *hma4* (**D**) genes in the shoots of soil-grown NaCl-untreated (−NaCl) and salt-stressed (+NaCl) *Mesembryanthemum crystallinum* plants subjected to concentrations of 0.01, 0.1, 1, 10 mM Cd and control (0.0 mM). Different letters above the bars indicate statistically significant differences at *p* ≤ 0.05 by Duncan’s post hoc test (*N* = 4, mean value ± SD).

Phytochelatin synthase 1 (*pcs1*) expression found in the roots of the control (Cd-, NaCl-untreated) plants was lower compared to the salt-stressed plants (Figure 2C). Upregulation of *pcs1* in roots of NaCl-untreated (−NaCl) plants was observed for all Cd treatments. In the case of the salt-stressed plants, Cd-induced *pcs1* expression in all tested treatments. One can note that a similar cumulative effect of both stressors was observed for *irt2*, as described above. In both tested plant groups, the upregulation effect was not concentration-dependent, and the most pronounced induction was found at 0.1 mM Cd in −NaCl plants and 0.01 and 10 mM Cd in +NaCl plants. The regulation of *pcs1* expression in shoots was affected differently by the applied stressors. No significant differences in the *pcs1* expression between shoots of untreated −NaCl and +NaCl plants, were found (Figure 3A). Upon Cd treatment of NaCl-untreated plants, the gene expression was upregulated only in the cases of 0.1 and 1 mM Cd. Concentration-dependent upregulation of *pcs1* expression was observed in shoots of salt-stressed plants subjected to up to 0.1 mM Cd.

ATPase-binding cassette 2 (*abcc2*) gene expression levels measured in the roots of the control (Cd-, NaCl-untreated plants) and salt-stressed plants differed insignificantly (Figure 2D). In −NaCl plants, treatment with 0.01 mM Cd resulted in the downregulation of *abcc2*. Application of 0.1 and 1 mM did not affect the expression of the gene. At the highest Cd concentration (10 mM), *abcc2* was upregulated. On the other hand, in the roots of the salt-stressed plants, Cd application induced random changes that were not correlated with increasing heavy metal concentrations: 0.01 and 10 mM Cd treatment led to *abcc2* upregulation, while 0.1 and 1 mM did not affect the expression of this gene. We found no significant differences in the shoot *abcc2* expression levels of the −NaCl (control) and salt-stressed (+NaCl) plants (Figure 3B). In the case of the NaCl-untreated plants, the gene expression was slightly modified only in response to 0.1 and 10 mM Cd concentrations. In turn, in the shoots of salt-stressed plants, all the treatments, except 0.1 mM Cd, significantly upregulated *abcc2* expression.

As in the case of the *hma4* and *pcs1* genes, the *cax4* (cation exchanger 4) expression found in roots of the Cd-untreated, salt-stressed plants was higher in comparison to controls (Figure 2E). None of the applied Cd concentrations enhanced the *cax4* expression, as established in the salt-stressed roots. On the other hand, in the roots of the −NaCl plants, all Cd treatments upregulated *cax4*. We found no difference in the shoot *cax4* expression of control (Cd-untreated, NaCl-untreated plants) and salt-stressed plants (Figure 3C). Cd treatments caused concentration-independent alterations of *cax4* expression in the shoots of both plant groups. The gene was upregulated by 0.01 and 0.1 mM Cd in NaCl-untreated plants and by 0.01 mM in salt-stressed plants.

In the roots of the salt-stressed ice plants, we found a significantly higher *hma4* (heavy metal ATPase 4) expression compared to the control (Cd-untreated, NaCl-untreated plants) (Figure 2F). Cd treatments did not modify the *hma4* expression established in plant roots after salt stress, except for the highest concentration, which resulted in a significant gene upregulation. In the NaCl-untreated plants, the Cd application starting with the lowest applied concentration induced *hma4* to the level found for the salt-stressed plants. The *hma4* expression determined in the shoots of salt-stressed plants was lower than that observed for the control (Figure 3D). Cd treatments caused downregulation of the gene in most of the examined variants. *hma4* upregulation was observed in both tested groups only in response to the highest Cd treatment (10 mM), and the effect was more pronounced for the NaCl-untreated plants.

## 3. Discussion

Essential heavy metals, e.g., Zn or Fe, are vital for plants to achieve intact growth and development, while the involvement of their non-essential relatives, i.e., Hg and Cd, in these processes is doubtful at best. Cadmium uptake from the soil is performed predominantly in the form of divalent cations and to some extent as chelates [15]. IRT1 and IRT2, as well as their respective orthologues, are ZIP family members. As divalent transporters, these proteins are responsible mostly for the uptake of Fe; however, their involvement in the trafficking of non-essential metals, such as Cd, was confirmed [24,25,26]. On the other hand, insufficient Fe levels promote Cd influx and accumulation in plants, while Fe supply initiates IRT1-mediated Cd uptake [27,28]. Based on the soil substrate analysis, for concentrations up to 1 mM, the amount of bioavailable Cd was very low. Despite this finding, the salt-stressed plants somehow sensed the introduced heavy metal which, in our opinion, was reflected by the induced *irt2* expression already observed at the lowest Cd treatment. It was recently discovered that *irt1* and *irt2* expression was amplified in glycophytic green grams (*Vigna radiata*) under Cd treatment in Fe-sufficient conditions [29]. In this study, we observed that salt stress upregulated *irt2* expression, and further Cd application enhanced this effect. The lack of Cd-triggered upregulation of *irt2* found in the NaCl-untreated plants could suggest that for halophytes, salt stress is required for Cd-induced modulation of *irt2* expression. Moreover, the observed interplay between salinity stress and *irt2* transcription regulation suggests an upstream position of salt stress in the *irt2* expression regulation pathway. The rate of Cd and Fe uptake *via* IRT transporters is orchestrated with a complex regulatory system, in which bioavailability seems to play an important role. This property is a result of antagonistic interactions recognized between both metals. For example, Cd presence can interfere with Fe assimilation, and thus contribute to Fe deficiency, despite its availability in the soil [30]. It was earlier proposed that the Cd-induced enhanced expression of *irt2* found in green grams is a part of the mechanism that allows coping with elevated Cd concentrations through improved Fe uptake [29]. However, at high bioavailable Cd concentrations, the effectiveness of such a mechanism could be negligible. In our opinion, more Cd accumulated in the roots of plants exposed to salinity (+NaCl), in comparison to NaCl-untreated plants (−NaCl) found under 10 mM treatment, despite similar Cd bioavailabilities, which could result from enhanced *irt2* expression. On the other hand, unaltered *irt2* transcript levels observed in the NaCl-untreated (−NaCl) plants could be responsible for restricted Cd uptake and significantly lower root accumulation compared to salt-stressed (+NaCl) plants. In conclusion, in a model of semi-halophyte *M. crystallinum*, salt stress plays an important role in the regulation of *irt2* expression during the plant response to elevated Cd concentrations. Moreover, both the salt and Cd stress effects on *irt2* expression were cumulative. The involvement of other ZIP family members, e.g., ZIP4, in essential (Mn) and non-essential (Cd) metal trafficking was confirmed [31]. Transcriptional regulation of ZIP transporters under Cd or salinity stress has not been thoroughly elucidated. Recently, enhanced expression of *zip4* in response to elevated Cd concentrations was described for non-hyperaccumulating ecotypes of *Sedum alfredii* [32]. A parallel result was observed in our experiment for both the NaCl-untreated and salt-stressed plants. Interestingly, our findings suggest that for the examined model semi-halophyte, the scheme of *zip4* expression, contrary to *irt2*, was not affected to such an extent by salt stress. The enhanced *zip4* expression found in response to elevated Cd concentration in the NaCl-untreated plants implies that plant salinity stress was not required for Cd-induced modification of the *zip4* expression scheme. Moreover, contrary to *irt2* regulation, no cumulative effect was observed, and both stressors caused *zip4* expression upregulation independently. Although the influence of the two stress factors on *zip4* expression was confirmed in this study, the accumulated Cd amounts, especially in salt-stressed roots, suggest the existence of a more complex mechanism orchestrating heavy metal uptake.

Phytochelatins (PC) are a well-described group of metal-complexing glutathione (GSH) derivatives, whose synthesis is rapidly induced in response to heavy metals [33]. PC synthase (PCS), whose gene *pcs1* has been recognized in *A. thaliana* and yeast, is responsible for phytochelatin synthesis [34,35]. The roles of PC and, indirectly, PCS in non-essential heavy metal detoxification have been unequivocally indicated with *A. thaliana* PC-deficient *cad1* mutants (AtPCS1 mutation) hypersensitive to As, Cd and Hg [36,37]. The GSH pool and PC synthesis remain interconnected, which revealed enhanced PCS activity, along with increasing concentrations of exogenously applied GSH and Cd in the tobacco BY-2 cell line [38]. The alteration of the GSH pool in response to different abiotic stresses is a well-described phenomenon, related mostly to antioxidative protection [39]. Salinity stress-induced CAM is accompanied by a significant increase in the GSH pool in *Mesembryanthemum crystallinum* [40]. The results of our study suggest that salt stress could fortify PC synthesis, not only by elevating the GSH pool, but also through an enhanced expression of *pcs1*. The mentioned expression alteration, however, was organ-specific and pronounced, especially in the roots. Interestingly, Cd treatment affected the root *pcs1* expression in both the NaCl-untreated and salt-stressed plants, which resulted in even stronger gene expression in the latter group, and further confirmed the cumulative effect of both stressors on the *pcs1* regulatory pathway. In earlier studies, it was documented that overexpression of the *A. thaliana pcs1* gene enhanced Cd accumulation and tolerance in tobacco seedlings as a result of increased PC synthesis [41]. High levels of PC identified in the *Brassica napus* phloem sap [42], as well as highly expressed *pcs1* in the *A. thaliana* phloem-loading cells, support the involvement of the PC-PCS system in long-distance metal distribution [43]. According to the above-mentioned studies, one cannot exclude the involvement of upregulated *pcs1* expression in phloem-mediated Cd-PC complex translocation, since significant concentrations of Cd were measured in shoots of both the NaCl-untreated and salt-stressed plants at the highest applied Cd concentration. In summary, the results of this study suggest that applied abiotic stressors, namely, salt and Cd stress, can synergistically affect the regulatory pathway of *pcs1*, contributing to the enhanced detoxifying potential of salt-stressed halophytes.

The next stage of heavy metal management involves ATP-dependent sequestration of metal-PC complexes within the vacuoles. Translocation of metal complexes *via* the tonoplast is carried out mainly by ATP-binding cassette (ABC)-type membrane transporters. The role of two best-described *A. thaliana* ABCC-type transporters, namely, AtABCC1 and AtABCC2, in Cd and Hg detoxification through vacuolar sequestration was demonstrated earlier [44]. As in the case of previously described genes involved in heavy metal homeostasis, the influence of salt stress on *abcc2* expression regulation has not been fully elucidated. The results obtained in this study suggest that, in contrast to *pcs1* and *irt2*, salt stress did not participate in *abcc2* expression regulation of roots and shoots. The response of ABCC-type transporter genes to heavy metal stress is considerably better known. It was demonstrated that Cd treatment did not affect *A. thaliana abcc1* and *abbc2* gene expression [45]; however, it enhanced the *abcc3* transcript level. Recently, similar *abcc1* and *abcc2* responses to Cd were also confirmed for rapeseed and wheat [46,47]. Our findings agree with the above-mentioned findings and support the idea of the lack of heavy metal involvement in halophyte *abcc2* expression regulation. Taken together, the experimental data indicate that both salt stress and Cd treatment have a rather slight effect on the *abcc2* gene regulatory pathway.

In addition to ATP-dependent transporters, such as ABCC-type transporters, the vacuolar sequestration of cations relies on antiporters energized by V-H^+^-ATPases and V-PPases. Cation exchangers (CAX) are a family of such antiporters predominantly responsible for Ca^2+^ homeostasis, but are also involved in other divalent cation trafficking [48]. Among the six CAXs identified in *Arabidopsis thaliana*, CAX2 and CAX4 are strongly involved in vacuolar sequestration of Cd, thereby conferring heavy metal tolerance [49,50]. The response of monovalent cation exchangers to salt stress is a well-described phenomenon [51,52]. However, the behavior of divalent transporters, such as CAXs, during salinity stress has not been determined. This subject requires elucidation, since the strong induction of activity and increased content of V-H^+^-ATPase, the enzyme responsible for the accumulation of an electrochemical gradient exploited during cation transport, has been revealed in *M. crystallinum* during salinity stress [53,54]. According to [55], the *cax4* steady-state expression level of the shoot is very low compared to the root. In this study, we confirmed a small share of both salinity and Cd treatment in the regulation of shoot *cax4* expression. On the other hand, *cax4* expression in roots was enhanced by salt stress and Cd treatment independently. 

Heavy metals that became neutralized inside the protoplast, either as free cations or chelates, can be applied to the metal circulation system of xylem and phloem, respectively [43]. While the mechanism regarding phloem loading and circulation still requires elucidation, the involvement of heavy metal ATPases (HMAs) in Cd xylem loading leaves no doubt. Most HMAs are influx pumps involved in the trafficking of both essential and toxic heavy metals. These ATPases may confer heavy metal resistance, such as AtHMA4, for which dysfunctional mutants were shown to be sensitive to elevated Cd and Zn levels [56]. It was proved that AtHMA2 and AtHMA4 were extraordinary ATPases [57]. Contrary to other ATPases, these proteins act as efflux pumps responsible for Cd and Zn xylem loading, thereby establishing the root-to-shoot translocation of both heavy metals. Although AtHMA4 is expressed predominantly in the roots, some environmental factors can alter its expression in other plant organs. It was shown that in the shoots of the *A. thaliana* seedlings, *hma4* expression was upregulated in response to sodium chloride [58]. In contrast to the aforementioned study, salt stress downregulated and upregulated *hma4* in shoots and roots, respectively. On the other hand, Cd treatment applied in our experiment to the NaCl-untreated plants upregulated not only *hma4* but also *cax4*, *pcs1* and *zip4* in the roots. Interestingly, *hma4* upregulation, as observed either for NaCl- or Cd-treatments, was not reflected by a significant rate of Cd translocation, with the only exception being the 10 mM Cd supplementation. Taken together, the above data, analogous to the cases of *cax4* and *pcs1*, show that both salt and Cd stress enhanced *hma4* expression in an organ-specific manner. However, similar to *cax4*, but contrary to *irt2* and *pcs1* regulation, the effect of both stressors was not cumulative; hence, NaCl and Cd could independently establish root readiness for heavy metal translocation at the transcriptional level. A different approach, however, was found earlier in glycophytes representative. Namely, [59] reported that AtHMA4 root expression was downregulated upon exposure to Cd stress. A similar response was later observed in the shoots of *A. thaliana* seedlings [58]. On the other hand, in Cd hyperaccumulators, such as *Arabidopsis helleri* and *Thlaspi caerulescens,* the HMA4 constitutive expression in roots and shoots was significantly higher in comparison to their Cd-sensitive counterparts [60,61]. In summary, it is possible that halophytes may represent intermediate features between sensitive and hyperaccumulating plants.

## 4. Materials and Methods

### 4.1. Plant Cultivation and Cd Treatment

*Mesembryanthemum crystallinum* L. seeds were sown on soil substrate in a greenhouse under 300–350 µmol photons m^−2^ s^−1^ of photosynthetically active radiation (PAR), a 16/8 h day/night period (25 °C /17 °C temperature) and 60%/65% relative humidity (RH). For plant cultivation, the market-available universal soil substrate “Hawita Fruhstorfer type LD80” (Hawita Gruppe GmbH, Germany) was used. The “LD80” soil is a ready-to-use substrate made with peat (H4–H6 and H6–H8), clay and type dependent ingredients, pH value (CaCl_2_) of 5.9, and salinity (1.0 in g/L KCl). The substrate contained CaCO_3_ and a long-term delivery fertilizer, providing N, P, K and Mg in concentrations of 150, 150, 250 and 130 mg/L, respectively. Two weeks after sowing, each seedling with a fully developed second leaf pair was transferred to an individual 1.2-L round pot with dimensions of 100 mm height × 125 mm deep. Six-week-old plants were divided into two groups: the first was irrigated with water (NaCl-untreated, −NaCl), while the second one was irrigated with 0.4 M NaCl (salt-stressed, +NaCl) for 14 days. In the next step, 8-week-old plants of both mentioned groups were irrigated with 10 cm^3^ solution of 0 (control), 0.01, 0.1, 1 and 10 mM CdCl_2_ (Sigma Aldrich, Saint Louis, MO, USA) each day of the 8-day-long treatment. No leakage from pots was detected during Cd application. On the day after the last Cd treatment, four plants of each experimental variant were harvested (roots and shoots independently). The fourth pair of leaves of each plant were collected, immediately frozen in liquid nitrogen, ground and then stored at −80 °C for molecular analyses. The remaining shoots (including leaves) and roots were used for biometric and Cd concentration analyses.

### 4.2. Biometric Analysis

For growth parameter and tissue water content determination, plant organs were prepared according to the procedures described in [9], with a slight modification regarding desiccation temperature. Specifically, the collected root parts were gently rinsed with cold distilled water until the soil substrate was removed; the shoots were rinsed briefly, and the roots were blotted with filter papers. For biometric analyses, the fresh weight was measured immediately, and the dry weight was assessed after 48 h of desiccation in an oven at 105 °C. The tissue water content (TWC) was calculated on a dry weight basis as follows:

TWC (cm^3^ g^−1^ DW) = (FW − DW)/DW

where FW and DW are the fresh and dry weights, respectively.

### 4.3. Cadmium Concentration Analysis

The soil substrate remaining after plant harvest was collected and stored at 4 °C. Prior to the analysis, the substrate was dried for 48 h in an oven at 105 °C and sieved (2 mm). For determination of the substrate, the Cd content extraction method involving the use of 0.01 M CaCl_2_, as described in [62], was employed. Each extraction was performed on 270 mg of soil substrate. Determination of *M. crystallinum* root and shoot Cd concentrations was carried out according to the extraction method previously described by [63]. The Cd content within the substrate and plant organs was measured with the method employing Graphite Furnace-Atomic Absorption Spectrometry (GF-AAS, Thermo iCE3000, Waltham, MA, USA). All standards were purchased from Sigma Aldrich (Saint Louis, MO, USA). All the reagents used in the experiment were trace element grade.

### 4.4. RNA Preparation

Total RNA was extracted from ground *M. crystallinum* roots and shoots frozen in liquid nitrogen (three plants per sample) using a Total RNA Mini Kit (Bio-Rad, Hercules, CA, USA). RNA purity and quantity were determined using a Biospec-Nano (Shimadzu, Japan).

### 4.5. qPCR

An iScript cDNA synthesis kit (Bio-Rad, Hercules, CA, USA) was used for reverse transcription carried out on 1000 ng of total RNA after digestion with DNase I (DNA I Amplification Grade, Merck, US). During qPCR, the samples were labelled with SYBR Green (iQ™ SYBR^®^ Green Supermix, Bio-Rad, Hercules, CA, USA) fluorescent dye. For a single reaction, 10–15 ng of cDNA and 150 nM of gene-specific primers were used (Supplementary Table S1). To test amplification specificity, a dissociation curve was acquired by heating samples from 60 °C to 95 °C. As a housekeeping reference, ubiquitin was used. The reaction efficiency was tested by serial dilutions of cDNAs with gene-specific primers (Supplementary Figure S1). The expression was calculated from at least three reactions according to [64]. Plants not treated with NaCl (−NaCl) and irrigated with 0 mM Cd served as calibrators.

### 4.6. Statistical Analysis

The results were analysed with Statistica 13.3 (StatSoft, Kraków, Poland) statistical software. One-way ANOVA followed by a post hoc test was used to evaluate individual treatment effects at *p* ≤ 0.05.

## 5. Conclusions

Salinity and Cd stress-enhanced expression of the root genes *irt2*, *cax4*, *hma4*, *pcs1* and *zip4* was verified using the ice plant model. For *irt2* and *pcs1*, a cumulative effect of both stressors on gene expression was found. Moreover, the role of salinity stress as an upstream regulator in the halophyte *irt2* expression scheme was suggested. 

## Figures and Tables

**Table 1:** Weight of roots and shoots of soil-grown NaCl-untreated (−NaCl) and salt-stressed (+NaCl) *Mesembryanthemum crystallinum* plants subjected to the elevated concentrations of Cd and control.

Cadmium Concentration [mM]	Root DW [g/Plant]		Shoot DW [g/Plant]	
	−NaCl	+NaCl	−NaCl	+NaCl
0	0.29 ± 0.06a	0.18 ± 0.06abc	1.93 ± 0.13a	2.03 ± 0.58a
0.01	0.27 ± 0.05a	0.13 ± 0.01bc	1.90 ± 0.18a	2.20 ± 0.41a
0.1	0.20 ± 0.05abc	0.21 ± 0.09abc	1.97 ± 0.22a	2.29 ± 0.57a
1.0	0.26 ± 0.07a	0.25 ± 0.04ab	2.09 ± 0.33a	2.06 ± 0.33a
10.0	0.21 ± 0.03abc	0.12 ± 0.04c	1.80 ± 0.35a	1.97 ± 0.37a

Means within root/shoot columns followed by the same letters are not significantly different at *p* < 0.05 according to Duncan’s test (*N* = 4, mean value ± SD).

**Table 2:** Root and shoot water content of soil-grown NaCl-untreated (−NaCl) and salt-stressed (+NaCl) *M. crystallinum* plants subjected to the elevated concentrations of Cd and control.

Cadmium Concentration [mM]	Root Water Content [cm^3^ g^−1^ DW]		Shoot Water Content [cm^3^ g^−1^ DW]	
	−NaCl	+NaCl	−NaCl	+NaCl
0	11.78 ± 4.27ab	8.77 ± 2.41abc	22.27 ± 1.43a	14.54 ± 1.48b
0.01	8.97 ± 0.53abc	6.47 ± 1.03bc	23.10 ± 2.22a	15.10 ± 1.37b
0.1	6.79 ± 0.89bc	5.34 ± 0.28bc	22.49 ± 2.84a	15.37 ± 1.48b
1.0	6.94 ± 0.32bc	7.99 ± 0.94abc	22.70 ± 2.75a	15.46 ± 1.53b
10.0	7.23 ± 1.15abc	10.04 ± 2.55ab	23.24 ± 2.61a	14.98 ± 2.81b

Means within root/shoot columns followed by the same letters are not significantly different at *p* < 0.05 according to Duncan’s test (*N* = 4, mean value ± SD).

**Table 3:** Shoot-to-root dry weight (DW) ratio in soil-grown NaCl-untreated (−NaCl) and salt-stressed (+NaCl) *M. crystallinum* plants subjected to elevated Cd concentrations and control.

Cadmium Concentration [mM]	Shoot-to-Root (DW) Ratio	
	−NaCl	+NaCl
0	7.01 ± 1.54c	10.22 ± 1.74bc
0.01	7.11 ± 0.84c	15.51 ± 3.75ab
0.1	9.57 ± 1.75bc	11.13 ± 3.19b
1.0	7.58 ± 2.33c	8.57 ± 0.78c
10.0	7.44 ± 0.54c	16.88 ± 3.46ab

Means within columns followed by the same letters are not significantly different at *p* < 0.05 according to Duncan’s test (*N* = 4, mean value ± SD).

## Supplementary Materials

The following are available online at https://www.mdpi.com/2223-7747/9/4/475/s1, Figure S1. Cadmium concentrations measured on the 1st, 3rd and 8th day of the experiment in the substrate of soil-grown NaCl-untreated (−NaCl) and salt-stressed (+NaCl) Mesembryanthemum crystallinum plants; Table S1. Sequences of primers employed for quantitative analysis of gene expression.

## Abbreviations

ABCC: ATPase-binding cassette type c; CAM, Crassulacean acid metabolism; CAX, cation exchanger; GF-AAS, Graphite Furnace-Atomic Absorption Spectrometry; HM, heavy metal; HMA, heavy metal ATPase; IRT, iron-regulated transporter; PCS, phytochelatin synthase; TWC, tissue water content; ZIP, ZRT IRT-like Protein; ZRT, zinc-regulated protein.
