# Supplementary material for: Expression of Genes Involved in Heavy Metal Trafficking in Plants Exposed to Salinity Stress and Elevated Cd Concentrations
Source: Plants (Basel). 2020 Apr 9;9(4):475. doi: 10.3390/plants9040475 (PMC7238198; doi:10.3390/plants9040475)
Supplement: Supplementary file 1 [file plants-09-00475-s001.zip › suplementary table T1.pdf]

Sequences of primers used during qPCR analysis

Name	Accession number	Sequence (5'–3')
ABCC2 – ATP-BINDING CASSETTE SUBFAMILY C	AT2G34660	F:AAACCGTTGGCTTGCTATCC<br>R:ACCTACCCGTTCAACAGCAT
CAX4 – CATION EXCHANGER	AT5G01490	F: TGGGCGATTGTGGTTCTACT<br>R: TCAGTCAACAGCTTCTCCCAT
HMA4 – HEAVY METAL ATPASE	AT2G19110	F:ACTGAAGCCACTTGAAGGAGT<br>R:CGCGAGCAATAACCCTGATA
IRT2 – IRON REGULATED TRANSPORTER	AT4G19680	F: CCATCTTCGATTTCCACCCG<br>R: GCGATTCAACCTCCACGTTT
PCS1 – PHYTOCHELATIN SYNTHASE	AT5G02190	F:AGCCAAGCATGCAACTTTCC<br>R:CCCCGCCATTGAATTTGCTT
UBQ – UBIQUITINE	AF053563	F: CGCACCTTGGCTGACTACA<br>R: AACAACCAGACCATGCAACA
ZIP4 – ZINC REGULATED PROTEIN	AT1G10970	F:ACGAGGATTGAAACGAGTGC<br>R:AAACCCCACCTTCGCTTTTC
