# Supplementary figures and images for: Expression of Genes Involved in Heavy Metal Trafficking in Plants Exposed to Salinity Stress and Elevated Cd Concentrations
Source: Plants (Basel). 2020 Apr 9;9(4):475. doi: 10.3390/plants9040475 (PMC7238198; doi:10.3390/plants9040475)

# Supplementary Fig. S1

**NaCl-  
untreated  
(-NaCl)**

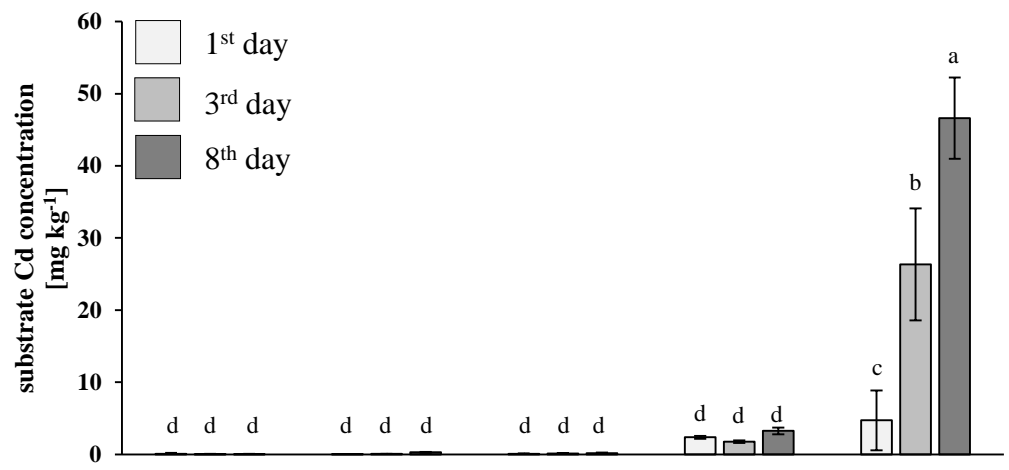

**salt-  
stressed  
(+NaCl)**

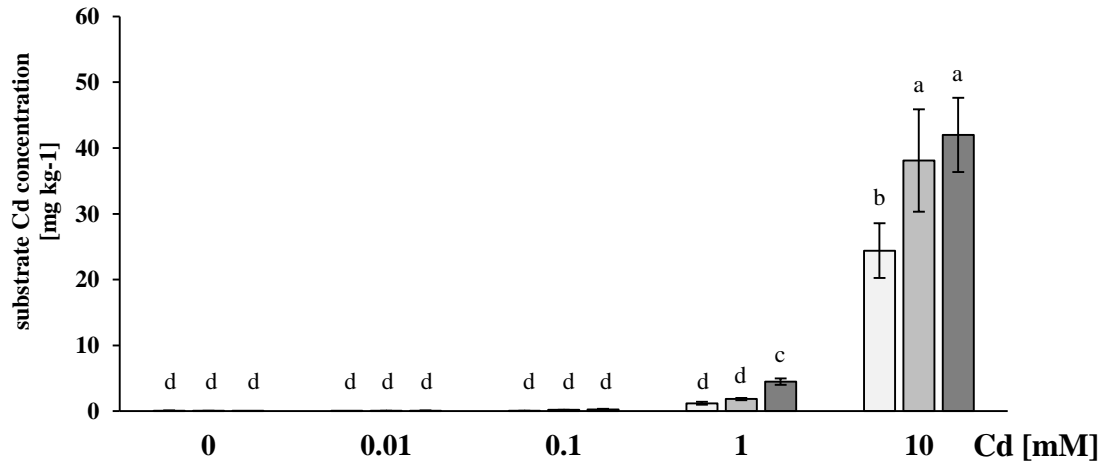

Supplement: Supplementary file 1 [file plants-09-00475-s001.zip › supplementary fig. S1.pdf]
